# Common variants in *KCNK5* and *FHL5* genes contributed to the susceptibility of migraine without aura in Han Chinese population

**DOI:** 10.1038/s41598-021-86374-0

**Published:** 2021-03-24

**Authors:** Zhao Jiang, Longrui Zhao, Xiaojie Zhang, Wenjuan Zhang, Yuxing Feng, Tao Li

**Affiliations:** 1 Department of Forensic Medicine, School of Medicine and Forensics, Xi’an Jiaotong University Health Science Center, 76 Yanta West Road, Xi’an, 710061 Shaanxi China; 2 Department of Neurology, Xijing Hospital of Air Force Medical University, Xi’an, Shaanxi China; 3 Department of Neurology, Xianyang Hospital of Yan’an University, Xi’an, Shaanxi China; 4 Department of Rehabilation and Pain Medicine, the Ninth People’s Hospital of Chongqing, Chongqing, China

**Keywords:** Biomarkers, Risk factors

## Abstract

A recent genome-wide meta study suggested that rs67338227 in the *FHL5* gene and rs10456100 in the *KCNK5* gene are associated with migraine from 27 population-based cohorts excluding Chinese population. Given that migraine without aura (MO) is the most common subtype of migraine, our aim was to systematically investigate the relationship of common variants in *FHL5* and *KCNK5* genes with the susceptibility to MO and provide clues as to the nature of the mechanisms involved in the etiology of migraine. A total of 3306 subjects including 1042 patients with MO and 2264 controls were recruited for the discovery stage, and 2530 individuals including 842 patients with MO and 1688 controls for the replication stage. Twenty-two tag SNPs (7 from *FHL5* and 15 from *KCNK5*) were selected for genotyping. Genetic associations were analyzed at both single-marker and haplotype levels. Potential functional consequences of the significant SNPs were analyzed using gene expression data obtained from the GTEx database. Two SNPs, rs10456100 (*KCNK5*, *P* = 9.01 × 10^–9^) and rs7775721 (*FHL5*, *P* = 6.86 × 10^–13^), were determined to be significantly associated with MO in the discovery sample and were then replicated in another sample. In the combined sample set, the T allele of both SNPs was significantly associated with the increased risk of MO. Significant eQTL signals were identified for both SNP rs10456100 and rs7775721. Our findings suggest that the T allele carriers of SNP rs10456100 and rs7775721 are at increased risk of migraine.

## Introduction

Migraine is a common neurovascular-disorder disease with 5%–15% incidence. As the third-highest cause of disability worldwide, severe migraine affects lives and results in serious economic consequences^1–3^. Depending on the clinical manifestations, migraine is characterized into two main types: migraine with aura (MA) and migraine without aura (MO)^4^. Because of the unclear etiology and pathogenesis of migraine, epidemiological studies on migraine are urgently needed. Moreover, developments of biotechnology and methods have contributed to new understanding of the migraine pathogenesis. There is a widely held belief that migraine development depends on the combined effect of genetic, vascular and nervous factors, family and twin studies have also demonstrated that the heritability of migraine is 30–60%, and the genetic contribution appears to differ among subtypes^5–8^.

Although knowledge of the underlying molecular mechanisms of migraine in the early stage is derived mainly from monogenic studies, considerable comprehensive genetic progress has been made in complex diseases currently based on the development of DNA sequencing techniques. In particular, genome-wide association studies (GWAS) and meta-analysis in large migraine cohorts recruiting millions of single nucleotide polymorphisms (SNPs) distributed across the genome have greatly helped to discover more genetic factors and pathways^9–11^. Comprehensive GWAS research results showed that positive migraine-related loci were particularly concentrated near genes involved in neuronal regulation and vascular functions^5^. Furthermore, neuronal regulations were affected by genes involving ion channels, glutamatergic transmission, nitric oxide or oxidative stress, or pain-sensing. Then, the ion channel *KCNK5* gene was identified as a migraine susceptibility locus. Furthermore, Olsen’ research suggested that the vessels dilate during MO and contract during MA^12^. Hence, vascular etiologies play significant roles in common migraine, and the genes implicated in vascular function could also be associated with migraine susceptibility, including the four-and-a-half LIM domains protein 5 (FHL5) gene. For its multiple polyadenylation sites, *FHL5* gene may stimulates vascular smooth muscle proliferation and migration via cAMP activation regulation to result in migraine.

A recent GWA meta study suggested that rs67338227 in the *FHL5* gene and rs10456100 in the *KCNK5* gene are associated with migraine from 27 population-based cohorts excluding Chinese population^10^. Then, Lin et al. found that the A allele of rs13208321 in *FHL5* gene was associated with reduced risk of migraine in a Chinese population^13^, suggesting that the *FHL5* gene is a potential risk gene for migraine. An et al. also performed a multilocus analysis to reveal the association between 17 candidate genes (including *FHL5* gene) and migraine susceptibility in Chinese population, but rs13208321 in *FHL5* gene showed no significant association signal for the susceptibility to migraine^14^. In addition, the relationship between *KNCK5* gene polymorphisms and the risk of migraine remains unknown. Given that MO is the most common subtype of migraine, we conducted this case–control study to evaluate the associations of *FHL5* and *KCNK5* genes with the risk of MO in a Han Chinese population. Our aim was to systematically investigate the relationship of common variants in *FHL5* and *KCNK5* genes with the susceptibility to MO and provide clues as to the nature of the mechanisms involved in the etiology of migraine.

## Methods

### Study subjects

We implemented a two-stage study design. In the discovery stage, a total of 22 SNPs were genotyped in 3306 study subjects. Significant SNPs identified in discovery stage were then genotyped in the independent samples of the replication stage comprising of 2530 study subjects. Subjects including 1042 patients with MO and 2264 healthy age-matched controls were recruited in the headache specialized clinic at the Department of Neurology of Xijing Hospital of Air Force Medical University and Xianyang Hospital of Yan’an University (Xi’an City). Subjects including 842 patients with MO and 1688 healthy age-matched controls were enrolled from the headache specialized clinic at the Department of Neurology of the Ninth People’s Hospital of Chongqing (Chongqing City). Two-stage subjects included in the study were unrelated Han Chinese individuals from Xi’an City and Chongqing City. All patients with MO were diagnosed by headache specialists based on the strict neurological examination results according to the criteria of the International Classification of Headache disorders (ICHD-3rd edition). Subjects with severe organic disease, tumors, neurological disorders, histories of depression or other comorbid psychiatric disorders involving migraines and nonmigrainous headaches were excluded. In addition, patients with secondary MO post-head injury were also excluded from the study. All healthy controls with no personal chronic headache were recruited from attendees at the physical examination centers in the hospitals mentioned above. Detailed demographic and medical history questionnaires were collected from all subjects, including age, gender, family history, unilateral pain, pulsating pain, nausea, vomiting, photophobia, phonophobia, and aggravation by physical activity. Written informed consent forms were obtained from all subjects. This study was carried out according to the ethical guidelines of the Declaration of Helsinki (version 2002) and was approved by the Medical Ethics Committee of Xi’an Jiaotong University Health Science Center.

### SNP selection and genotyping

SNPs located within gene regions of *FHL5* and *KCNK5* were extracted for genotyping. Based on 1000 genome Chinese Han Beijing (CHB) data, we selected 21 tag SNPs (from 162 SNPs with MAF > 0.05) for genotyping (6 from *FHL5* and 15 from *KCNK5*) using the criteria of r^2^ ≥ 0.5. In addition, SNP rs67338227 was also included for purposes of replication for a previous meta-study^10^. All 22 SNPs were located at intronic regions of the two genes (Supplemental Table S1). The peripheral blood samples of the study subjects were utilized for genomic DNA extractions based on the Tiangen DNA extraction kit (Tiangen Biotech Co. Ltd, Beijing, China). The Sequenom MassARRAY platform with iPLEX GOLD chemistry (Sequenom, San Diego, CA, USA) was used for SNP genotyping. To minimize artificial factors, laboratory personnel were blinded to labels of case and control for each sample during the experiments^15,16^. Data entry was also reviewed independently by two authors. Finally, 5% of the random sample was replicated for SNP genotyping, and the concordance rate was 100%.

### Statistical power analyses

We have utilized GAS power calculator (version 4.2.5, http://csg.sph.umich.edu/abecasis/gas_power_calculator/) to conduct the statistical power analyses. The parameters utilized for the power analyses were summarized in Supplemental Table S2. The results indicated that our study setting could achieve 82.8% statistical power in detecting a genetic association signal with the genotypic relative risk of 1.2 (Supplemental Figure S1).

### Statistical and bioinformatic analyses

Hardy–Weinberg equilibrium (HWE) was tested in controls. Single marker-based genetic associations were analyzed by performing χ^2^ tests. Linkage disequilibrium (LD) blocks were constructed and haplotype-based association analyses were conducted for each LD block. In addition, distributions of clinical variables for patients with migraine in genotype groups of the significant SNPs were also examined. Bonferroni corrections were applied to address multiple comparisons. The threshold of *P* values chosen for discovery stage was 0.05/22≈0.002. Plink^17^ and Haploview^18^ were utilized for genetic association analyses. R project was used for general statistical analyses^19^.

To examine the potential functional consequences of the significant SNPs, we extracted gene expression data from the GTEx database to investigate the tissue-specific expression quantitative traits loci (eQTL) and splicing quantitative trait loci (sQLT). The GTEx database (https://www.gtexportal.org/home/) is derived from the Genotype-Tissue Expression (GTEx) project that aims to collect and analyze multiple human tissues that are densely genotyped and analyzed for global RNA expression^20^.

## Results

### Demographic and clinical characteristics of the study subjects

A total of 3306 study subjects including 1042 patients with MO and 2264 controls were recruited for discovery stage. A total of 2530 individuals including 842 patients with MO and 1688 controls were enrolled for the replication stage. Age, gender and family history of migraine were compared between controls and patients with migraine in the discovery and replication samples, and no significant differences were identified (Table 1).

**Table 1:** Demographic and clinical information of the study subjects.

Clinical variables	Discovery stage (N = 3306)		Statistics	*P* value	Replication stage (N = 2530)		Statistics	*P* value
	Cases (N = 1042)	Controls (N = 2264)			Cases (N = 842)	Controls (N = 1688)		
Age, mean ± SD	37.8 ± 7.2	37.8 ± 7.4	t = 0.14	0.89	36.7 ± 7.1	36.8 ± 7.0	t = −0.34	0.73
**Gender (%)**								
Male	295 (28)	643 (28)			187 (22)	1310 (78)		
Female	747 (72)	1621 (72)	χ^2^ = 0.0001	0.99	655 (78)	378 (22)	χ^2^ = 0.0029	0.96
**Family history (%)**								
Yes	68 (7)	120 (5)			62 (7)	105 (4)		
No	974 (93)	2144 (95)	χ^2^ = 1.78	0.18	780 (93)	1583 (96)	χ^2^ = 1.01	0.31
**Unilateral pain (%)**								
Yes	792 (76)	–			690 (82)	–		
No	250 (24)	–	–	–	152 (18)	–	–	–
**Pulsating pain (%)**								
Yes	677 (65)	–			581 (69)	–		
No	365 (35)	–	–	–	261 (31)	–	–	–
**Nausea (%)**								
Yes	854 (82)	–			674 (80)	–		
No	188 (18)	–	–	–	168 (20)	–	–	–
**Vomiting (%)**								
Yes	771 (74)	–			598 (71)	–		
No	271 (26)	–	–	–	244 (29)	–	–	–
**Photophobia (%)**								
Yes	834 (80)	–			657 (78)	–		
No	208 (20)	–	–	–	185 (22)	–	–	–
**Phonophobia (%)**								
Yes	896 (86)	–			699 (83)	–		
No	146 (14)	–	–	–	143 (17)	–	–	–
**Aggrevation by physical activity (%)**								
Yes	969 (93)	–			758 (90)	–		
No	73 (7)	–	–	–	84 (10)	–	–	–

### Genetic association between MO and SNPs

**Figure 1:**
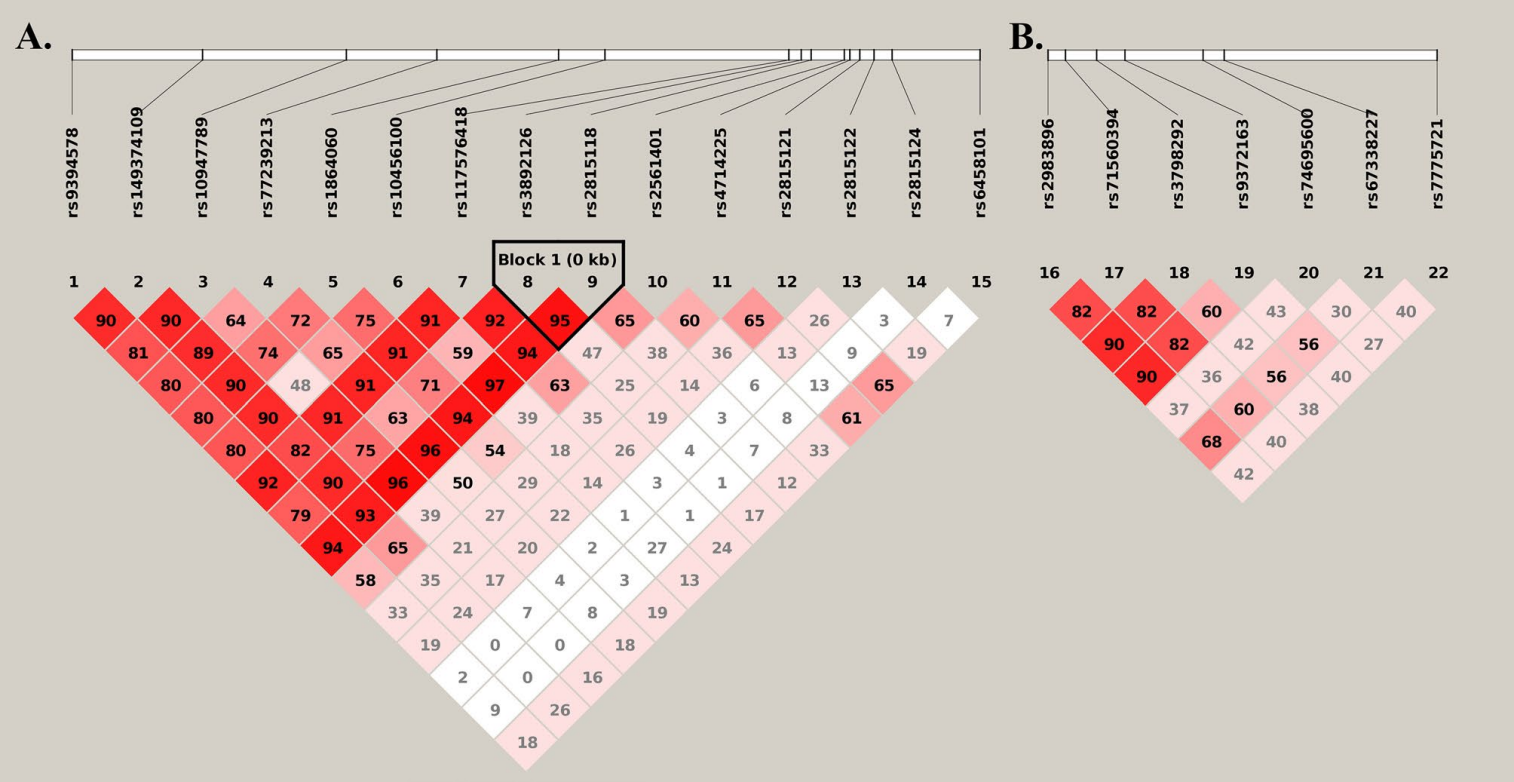
Linkage disequilibrium plots for gene *KCNK5* and *FHL5.* (**A**) Linkage disequilibrium plot for gene *KCNK5*; (**B**) Linkage disequilibrium plot for gene *FHL5*.

All 22 genotyped SNPs were in HWE in controls (Supplemental Table S1). Two SNPs were identified to be significantly associated with migraine in the discovery sample and were then replicated in the replication sample (Table 2). In the combined sample set, SNP rs10456100 (*KCNK5*) was significantly associated with migraine (χ^2^ = 37.05, *P* Value = 9.01 × 10^–9^, Table 2). The T allele of this SNP was significantly associated with the increased risk of migraine (OR_TT_ = 2.11[1.56–2.86], OR_CT_ = 1.29[1.15–1.46], Table 2). As shown in Table 2, SNP rs7775721 (*FHL5*) was also significantly associated with migraine (χ^2^ = 56.02, *P* value = 6.86 × 10^–13^) in the combined sample set, and its T allele was significantly associated with the increased risk of migraine (OR_TT_ = 1.85 [1.57–2.17], OR_CT_ = 1.25 [1.11–1.42]). Dose-dependence patterns could be observed for both SNPs. The full results of the single marker-based association analyses in discovery stage are summarized in Supplemental Table S3. LD plots for both genes were constructed (Fig. 1) and only one LD block was identified. It is a two-SNP LD block (rs3892126-rs2815118) located within gene region of *KCNK5*. No significant signals were identified in haplotype-based association analyses (Supplemental Table S4). For both significant SNPs, there is no significant difference for clinical variables in different genotype groups (Supplemental Table S5). To investigate the potential systematic inflation of association signals caused by population stratification, we generated Q-Q plots based on the results of allelic association analyses and the result is shown in Supplemental Figure S2. No systematic inflation for the association signals could be identified.

**Table 2:** Significant results for single marker based association analysis.

Sample set	SNP	Genotypic analysis						Allelic analysis^a^					
		Geno (%)	Cases (N = 1042)	Controls (N = 2264)	OR [95%CI]	χ^2^	*P*	Alleles (%)	Cases (N = 2084)	Controls (N = 4528)	OR[95%CI]	Z	*P*
DS	rs10456100	TT	46 (4)	52 (2)	–				–	–			
		CT	313 (30)	580 (26)	2.11 [1.41–3.17]			T	405 (19)	684 (15)			
		CC	683 (66)	1632 (72)	1.29 [1.09–1.52]	20.3	3.9 × 10^–5^	C	1679 (81)	3844 (85)	1.34 [1.21–1.49]	5.4	5.6 × 10^–8^
	rs7775721	TT	206 (20)	299 (13)	–				–	–			
		CT	496 (48)	1055 (47)	1.84 [1.48–2.29]			T	908 (44)	1653 (37)			
		CC	340 (32)	910 (40)	1.26 [1.07–1.48]	31.1	1.8 × 10^–7^	C	1176 (56)	2875 (63)	1.35 [1.18–1.54]	4.4	1.4 × 10^–5^

			Cases (N = 842)	Controls (N = 1688)					Cases (N = 1684)	Controls (N = 3376)			
RS	rs10456100	TT	39 (5)	41 (2)	-				-	-			
		CT	254 (30)	432 (26)	2.11 [1.34–3.30]			T	332 (20)	514 (15)			
		CC	549 (65)	1215 (72)	1.30 [1.08–1.57]	16.7	2.4 × 10^–4^	C	1352 (80)	2862 (85)	1.36 [1.17–1.58]	4.0	7.3 × 10^–5^
	rs7775721	TT	168 (20)	223 (13)	-				-	-			
		CT	397 (47)	783 (46)	1.85 [1.45–2.37]			T	733 (44)	1229 (36)			
		CC	277 (33)	682 (41)	1.25 [1.04–1.50]	24.9	3.8 × 10^–6^	C	951 (56)	2147 (64)	1.34 [1.19–1.51]	4.8	1.3 × 10^–6^

			Cases (N = 1884)	Controls (N = 3952)					Cases (N = 3768)	Controls (N = 7904)			
CS	rs10456100	TT	85 (5)	93 (2)	–				–	–			
		CT	567 (30)	1012 (26)	2.11 [1.56–2.86]			T	737 (20)	1198 (15)			
		CC	1232 (65)	2847 (72)	1.29 [1.15–1.46]	37.1	9.0 × 10^–9^	C	3031 (80)	6706 (85)	1.35 [1.22–1.50]	5.9	3.9 × 10^–9^
	rs7775721	TT	374 (20)	522 (13)	–				–	–			
		CT	893 (47)	1838 (47)	1.85 [1.57–2.17]			T	1641 (44)	2882 (36)			
		CC	617 (33)	1592 (40)	1.25 [1.11–1.42]	56.0	6.9 × 10^–13^	C	2127 (56)	5022 (64)	1.34 [1.24–1.45]	7.3	3.4 × 10^–13^

DS: discovery stage; RS: replication stage; CS: combined set. Geno: Genotypes.

Z: Z-statistics.

^a^Logistic models were fitted for allelic analysis. OR with 95% confidence intervals and *P* values were adjusted by age and gender.

### Significant eQTL and sQTL signals captured on rs10456100 and rs7775721

**Figure 2:**
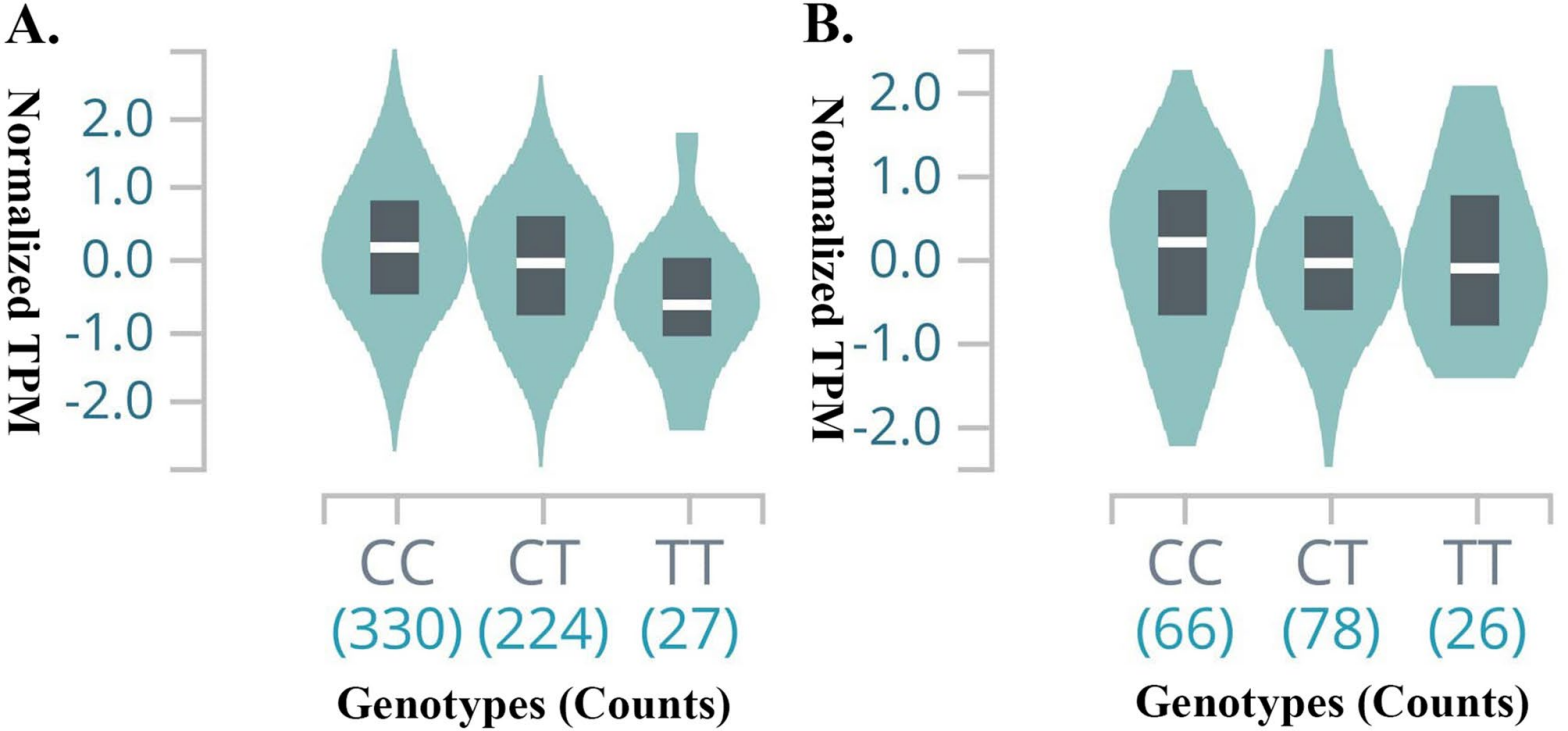
Violin plots for genotypes of SNP rs10456100 and rs7775721 on gene expression level of *KCNK5* and *FHL5*. (**A**) Violin plot for genotypes of SNP rs10456100 on *KCNK5* gene expression levels in human tissue of subcutaneous adipose; (**B**) Violin plot for genotypes of SNP rs7775721 on *FHL5* gene expression levels in human tissue of brain putamen.

Significant eQTL signals were identified for both SNP rs10456100 and rs7775721 (Supplemental Table S6 and S7). The threshold of *P* values was 0.05/46 = 0.001. The T allele of rs10456100 was significantly associated with decreased gene expression level of *KCNK5* in subcutaneous adipose tissue (Fig. 2A, *P* = 2.70 × 10^–10^). On the other hand, the T allele of rs7775721 was significantly associated with decreased gene expression of *FHL5* in the putamen of the brain (Fig. 2B, *P* = 2.70 × 10^–10^). In addition, we identified widespread significant sQTL signals for rs7775721 on *FHL5* (Table 3). The T allele of rs7775721 was related to the decreased level of alternative splicing of the pre-mRNA for gene *FHL5* from multiple types of human tissues.

**Table 3:** Significant sQTL signals of SNP rs7775721 on *FHL5*.

Gene	Ref_Alt Alleles	SNP	*P* value	NES	Tissue
*FHL5*	C_T	rs7775721	3.40 × 10^–59^	− 0.9	Artery—Tibial
*FHL5*	C_T	rs7775721	8.50 × 10^–37^	− 0.73	Adipose—Subcutaneous
*FHL5*	C_T	rs7775721	1.70 × 10^–19^	− 0.57	Nerve—Tibial
*FHL5*	C_T	rs7775721	3.10 × 10^–19^	0.51	Testis
*FHL5*	C_T	rs7775721	3.30 × 10^–17^	− 0.57	Adipose—Visceral (Omentum)
*FHL5*	C_T	rs7775721	7.60 × 10^–15^	− 0.49	Skin—Sun Exposed (Lower leg)
*FHL5*	C_T	rs7775721	2.10 × 10^–14^	− 0.5	Thyroid
*FHL5*	C_T	rs7775721	6.90 × 10^–14^	− 0.55	Artery—Aorta
*FHL5*	C_T	rs7775721	3.00 × 10^–13^	0.48	Lung
*FHL5*	C_T	rs7775721	5.40 × 10^–13^	− 0.55	Breast—Mammary Tissue
*FHL5*	C_T	rs7775721	5.60 × 10^–13^	− 0.73	Artery—Coronary
*FHL5*	C_T	rs7775721	7.90 × 10^–10^	− 0.46	Esophagus—Muscularis
*FHL5*	C_T	rs7775721	1.10 × 10^–6^	− 0.44	Esophagus—Gastroesophageal Junction
*FHL5*	C_T	rs7775721	1.20 × 10^–6^	− 0.39	Heart—Left Ventricle
*FHL5*	C_T	rs7775721	4.90 × 10^–6^	− 0.28	Muscle—Skeletal
*FHL5*	C_T	rs7775721	6.10 × 10^–6^	− 0.33	Esophagus—Mucosa

Ref_Alt Alleles: reference and alternative alleles.

## Discussion

In the present study, we identified two significant genetic association signals, SNP rs10456100 in *KCNK5* and SNP rs7775721 in *FHL5*, of MO in a Chinese Han population. For SNP rs10456100, although a recent large-scale GWA meta-study reported this association signal, a Chinese population was not included in that study^10^. In this sense, our study could be considered a successful replication of this previous study in a Chinese population. The effect direction of SNP rs10456100 was the same for both studies, even though the effect size was considerably greater in the present study. Furthermore, SNP rs67338227 was also reported to be significant in the GWA meta-study. However, in the present study, we did not identify this signal. In addition, another study based on European population indicated that SNP rs9394578 in *KCNK5* was a significant association signal for risk of MO^21^. However, this association signal was also not replicated in our Chinese samples. These inconsistencies could be explained, at least in part, by differences in genetic background of the study populations. For example, the minor allele frequency (the A allele) of SNP rs9394578 was 0.23 in a European population (1000 genome data) compared to 0.08 in our Chinese Han data.

For SNP rs7775721, to the best of our knowledge, no previous study has reported this genetic association signal, although multiple genetic association studies have been conducted for genetic polymorphisms of *FHL5* and risk of MO. SNP rs2983896 was identified to be significantly associated with risk of MO in a study based on European population in 2016^22^; however, this genetic association signal was not replicated in the present study. Similar to the association signals in *KCNK5*, we believe that this inconsistency could also be explained by the genetic heterogeneity of different populations. In addition, at least two previous studies have investigated the associations between genetic polymorphisms in *FHL5* and risk of MO based on Chinese populations^13,14^. SNP rs13208321 was found to contribute to the risk of MO in both studies. In the present study, this SNP was not included because it was located out of the gene region of *FHL5*.

It is beyond the scope of this study to investigate the pathological significance of the association signals. However, we may still conduct a general discussion based on previous studies and the present one. In general, association signals between an SNP and a disorder could follow one of the two following situations. The first is that this SNP might have true effects on the pathological mechanisms of the disorder. Another is that this SNP is a surrogate of some underlying ungenotyped DNA variant that has true effects on the onset and development of the disorder. One thing interesting to note is that, for both loci, multiple different SNPs have been reported to be associated with MO. Therefore, it is probable that both SNP rs10456100 in *KCNK5* and SNP rs7775721 in *FHL5* might only be surrogates without true effects. With the widespread application of high-throughput technology, many susceptible genes were identified to be contributed to the risk of complex diseases^23–25^, but it is difficult to draw solid conclusions only from SNP results^26–28^. Thus, more research on the genetic architecture of the two loci are still needed in the future to further investigate the pathological significance of the two association signals.

The *KCNK5* gene is located at 6p21.2 with 5 exons. The protein encoded by *KCNK5* gene is a member of the super family K of potassium channel proteins. Multiple lines of evidence have shown that other members of the super family K are also indicated to be linked with migraine susceptibility^29^. K^+^ plays an important role in a variety of metabolic activities, and it has to get into the cytoplasm via potassium channels first before its functional implementation. Therefore, *KCNK5* gene possibly regulates the K^+^-related membrane potential and excitability to induce the occurrence and development of migraine^30^. Moreover, high extracellular K^+^ can cause vascular smooth muscle contraction, vasospasm, ischemia, and cortex neuron activity inhibition, which in turn leads to migraine^31,32^. Interestingly, in the present study, we found that the T allele of SNP rs10456100 was associated with increased risk of migraine and decreased level of *KCNK5* gene expression. Based on these findings, we hypothesize that this allele might increase extracellular K^+^ levels by reducing the transcription of the *KCNK5* gene, in turn increasing the risk of migraine for individuals. More studies in future are still needed to investigate the underlying mechanisms of *KCNK5* gene and pathology of migraine.

The *FHL5* gene is located at 6q16.1 with 8 exons. The protein encoded by *FHL5* gene is coordinately expressed with activator of cAMP-responsive element modulator (CREM) and confers a powerful transcriptional activation function. In the present study, we showed that the T allele of SNP rs7775721 was associated with increased risk of MO and decreased gene expression level of *FHL5*. In addition, bioinformatics evidence also indicated that the T allele of rs7775721 was related to decreased levels of alternative splicing of the pre-mRNA for gene *FHL5* from multiple types of human tissues. Nevertheless, it has not been determined how to connect the functional consequences of rs7775721 and the pathology of migraine. Additional studies should be conducted for *FHL5* gene to unravel the underlying pathological mechanisms.

This study suffers from several limitations. As a candidate gene-based study, it is difficult for us to adjust for population stratifications. Although the Q-Q plot showed no significant inflation of these association signals, we still cannot rule out the possibility that the significant findings might be spurious. In addition, we investigated the potential functional consequences of SNP rs10456100 and SNP rs7775721 through eQTL and sQTL data obtained from public available database. However, we need to be careful to interpret these data because samples collected by the GTEx database are not patients with MO. Gene expression patterns could be quite different in tissues of patients with migraine compared to those of healthy individuals.

In conclusion, our findings indicated that the T allele carriers of SNP rs10456100 and rs7775721 would increase the risk of MO in a Han Chinese population, providing evidence indicating that the *KCNK5* and *FHL5* genes contribute to the susceptibility of migraine. These findings help to elucidate the pathogenesis of migraine.

## Supplementary Information

Supplementary information.
