# Supplementary material for: Common variants in KCNK5 and FHL5 genes contributed to the susceptibility of migraine without aura in Han Chinese population
Source: Sci Rep. 2021 Mar 24;11:6807. doi: 10.1038/s41598-021-86374-0 (PMC7990926; doi:10.1038/s41598-021-86374-0)
Supplement: Supplementary file 1 — Supplementary information. [file 41598_2021_86374_MOESM1_ESM.docx]

***Title:*** Common variants in *KCNK5* and *FHL5* genes contributed to the susceptibility of migraine without aura in Han Chinese population

***Running title*:** *KCNK5* and *FHL5* genes and migraine without aura

***Author names and affiliations***: Zhao Jiang ^1,2*^, Longrui Zhao ^1*^, Xiaojie Zhang ^3^, Wenjuan Zhang ^2^, Yuxing Feng ^4^ and Tao Li ^1^

^1^ Department of Forensic Medicine, School of Medicine & Forensics, Xi’an Jiaotong University Health Science Center, Xi’an, Shaanxi, China;

^2^ Department of Neurology, Xijing Hospital of Air Force Medical University, Xi’an, Shaanxi, China;

^3^ Department of Neurology, Xianyang Hospital of Yan’an University, Xi’an, Shaanxi, China;

^4^ Department of Rehabilation and Pain Medicine, the Ninth People’s Hospital of Chongqing, Chongqing, China.

* These authors contributed equally to this work.

***Corresponding Author***:

Tao Li, M.D. & Ph.D., Department of Forensic Medicine, School of Medicine & Forensics, Xi’an Jiaotong University Health Science Center, 76 Yanta West Road, Xi’an, Shaanxi, China, 710061.

Tel: 86-29-82655117; Fax: 86-29-82655472.

E-mail: litao050428@mail.xjtu.edu.cn

Supplemental Table S1. Genetic information for the 22 SNPs selected for genotyping.

| CHR | Position | SNP | Gene | Function | A1 | A2 | MAF | HWE |
| --- | --- | --- | --- | --- | --- | --- | --- | --- |
| 6 | 39198083 | rs9394578 | *KCNK5* | intron | A | C | 0.09 | 0.89 |
| 6 | 39202392 | rs149374109 | *KCNK5* | intron | T | A | 0.07 | 0.60 |
| 6 | 39207146 | rs10947789 | *KCNK5* | intron | C | T | 0.18 | 0.67 |
| 6 | 39210138 | rs77239213 | *KCNK5* | intron | A | C | 0.11 | 0.76 |
| 6 | 39214162 | rs1864060 | *KCNK5* | intron | T | G | 0.10 | 0.72 |
| 6 | 39215694 | rs10456100 | *KCNK5* | intron | T | C | 0.16 | 0.93 |
| 6 | 39221773 | rs117576418 | *KCNK5* | intron | C | T | 0.07 | 0.74 |
| 6 | 39222183 | rs3892126 | *KCNK5* | intron | G | C | 0.13 | 0.77 |
| 6 | 39222511 | rs2815118 | *KCNK5* | intron | T | A | 0.38 | 0.79 |
| 6 | 39223608 | rs2561401 | *KCNK5* | intron | C | T | 0.24 | 0.86 |
| 6 | 39223790 | rs4714225 | *KCNK5* | intron | A | G | 0.21 | 0.85 |
| 6 | 39224118 | rs2815121 | *KCNK5* | intron | G | A | 0.38 | 0.72 |
| 6 | 39224590 | rs2815122 | *KCNK5* | intron | T | C | 0.15 | 0.87 |
| 6 | 39225186 | rs2815124 | *KCNK5* | intron | T | C | 0.06 | 0.55 |
| 6 | 39228095 | rs6458101 | *KCNK5* | intron | T | A | 0.42 | 0.97 |
| 6 | 96581995 | rs2983896 | *FHL5* | intron | A | G | 0.07 | 0.74 |
| 6 | 96583211 | rs71560394 | *FHL5* | intron | T | C | 0.08 | 0.58 |
| 6 | 96585385 | rs3798292 | *FHL5* | intron | G | A | 0.15 | 0.93 |
| 6 | 96587355 | rs9372163 | *FHL5* | intron | G | A | 0.13 | 0.85 |
| 6 | 96592798 | rs74695600 | *FHL5* | intron | A | G | 0.06 | 0.36 |
| 6 | 96594271 | rs67338227 | *FHL5* | intron | T | A | 0.09 | 0.90 |
| 6 | 96609103 | rs7775721 | *FHL5* | intron | T | C | 0.39 | 0.82 |

A1: minor allele; A2: major allele; MAF: minor allele frequency; HWE: *P*-value for Hardy Weinberg equilibrium tests conducted in controls.

Supplemental Table S2. Parameters chosen for statistical power analyses.

| Parameter | Value | Note |
| --- | --- | --- |
| Cases | 1,884 | Sample size |
| Controls | 3,952 | Sample size |
| Significance Level | 0.002 | 0.05/22≈0.002 |
| Prevalence | 0.15 | Vos T *et al.* *Lancet.* 380 (9859): 2163–96. |
| Disease Allele Frequency | 0.17 | Average MAF of the 22 selected SNPs |
| Genotype Relative Risk | 1.2 | - |

Supplemental Table S3. Full results for singe maker based association analysis in the discovery stage

| CHR | SNP | Gene | A1 | A2 | *AFF | *UNAFF | χ^2^ | DF | *P*-Value |
| --- | --- | --- | --- | --- | --- | --- | --- | --- | --- |
| 6 | rs9394578 | *KCNK5* | A | C | 9/171/862 | 16/344/1904 | 1.07 | 2 | 0.59 |
| 6 | rs149374109 | *KCNK5* | T | A | 8/129/905 | 11/272/1981 | 1.10 | 2 | 0.58 |
| 6 | rs10947789 | *KCNK5* | C | T | 37/311/694 | 68/670/1526 | 0.76 | 2 | 0.69 |
| 6 | rs77239213 | *KCNK5* | A | C | 9/213/820 | 28/466/1770 | 0.92 | 2 | 0.63 |
| 6 | rs1864060 | *KCNK5* | T | G | 8/181/853 | 23/394/1847 | 0.48 | 2 | 0.79 |
| 6 | rs10456100 | *KCNK5* | T | C | 46/313/683 | 52/580/1632 | 20.31 | 2 | 3.88×10^-5^ |
| 6 | rs117576418 | *KCNK5* | C | T | 8/135/899 | 11/281/1972 | 1.21 | 2 | 0.55 |
| 6 | rs3892126 | *KCNK5* | G | C | 20/229/793 | 36/489/1739 | 0.55 | 2 | 0.76 |
| 6 | rs2815118 | *KCNK5* | T | A | 142/498/402 | 322/1073/869 | 0.21 | 2 | 0.90 |
| 6 | rs2561401 | *KCNK5* | C | T | 56/377/609 | 131/820/1313 | 0.24 | 2 | 0.89 |
| 6 | rs4714225 | *KCNK5* | A | G | 48/352/642 | 95/747/1422 | 0.57 | 2 | 0.75 |
| 6 | rs2815121 | *KCNK5* | G | A | 149/490/403 | 312/1069/883 | 0.16 | 2 | 0.92 |
| 6 | rs2815122 | *KCNK5* | T | C | 19/258/765 | 50/566/1648 | 0.56 | 2 | 0.76 |
| 6 | rs2815124 | *KCNK5* | T | C | 5/113/924 | 9/240/2015 | 0.16 | 2 | 0.92 |
| 6 | rs6458101 | *KCNK5* | T | A | 177/506/359 | 394/1100/770 | 0.11 | 2 | 0.95 |
| 6 | rs2983896 | *FHL5* | A | G | 9/140/893 | 11/281/1972 | 2.43 | 2 | 0.30 |
| 6 | rs71560394 | *FHL5* | T | C | 7/148/887 | 13/349/1902 | 0.91 | 2 | 0.63 |
| 6 | rs3798292 | *FHL5* | G | A | 25/261/756 | 46/561/1657 | 0.51 | 2 | 0.78 |
| 6 | rs9372163 | *FHL5* | G | A | 20/246/776 | 36/512/1716 | 0.93 | 2 | 0.63 |
| 6 | rs74695600 | *FHL5* | A | G | 8/131/903 | 11/258/1995 | 2.00 | 2 | 0.37 |
| 6 | rs67338227 | *FHL5* | T | A | 13/188/841 | 18/362/1884 | 3.91 | 2 | 0.14 |
| 6 | rs7775721 | *FHL5* | T | C | 206/496/340 | 299/1055/910 | 31.07 | 2 | 1.79×10^-7^ |

CHR: chromosome; A1:minor allele; A2:major allele; AFF: counts of individuals for each genotype in cases; UNAFF: counts of individuals for each genotype in controls; DF: degree of freedom.

*A1A1/A1A2/A2A2.

Supplemental Table S4.Results for haplotype based association analysis.

| LOCUS | HAPLOTYPE | F_A | F_U | χ^2^ | DF | *P*-Values | SNPs |
| --- | --- | --- | --- | --- | --- | --- | --- |
| H1 | OMNIBUS | - | - | 0.34 | 2 | 0.84 | rs3892126\|rs2815118 |
| H1 | GT | 0.12 | 0.12 | 0.13 | 1 | 0.72 | rs3892126\|rs2815118 |
| H1 | CT | 0.25 | 0.26 | 0.28 | 1 | 0.59 | rs3892126\|rs2815118 |
| H1 | CA | 0.62 | 0.62 | 0.06 | 1 | 0.81 | rs3892126\|rs2815118 |

F_A: haplotype frequency in cases; F_U: haplotype frequency in controls; DF: degree of freedom.

Supplemental Table S5. Association between significant SNPs and clinical variables in migraine cases.

| Clinical Variables | rs7775721 | | | Statistics | *P*-Values | rs10456100 | | | Statistics | *P*-Values |
| --- | --- | --- | --- | --- | --- | --- | --- | --- | --- | --- |
|  | TT (N=374) | CT (N=893) | CC (N=617) |  |  | TT (N=85) | CT (N=567) | CC (N=1,232) |  |  |
| Unilateral Pain (%) |  |  |  |  |  |  |  |  |  |  |
| *Yes* | 300 (80) | 681 (76) | 501 (81) |  |  | 65 (76) | 452 (80) | 965 (78) |  |  |
| *No* | 74 (20) | 212 (24) | 116 (19) | χ^2^ = 5.97 | 0.0505 | 20 (24) | 115 (20) | 267 (22) | χ^2^ = 0.70 | 0.7041 |
| Pulsating Pain (%) |  |  |  |  |  |  |  |  |  |  |
| *Yes* | 250 (67) | 599 (67) | 409 (66) |  |  | 53 (62) | 375 (66) | 830 (67) |  |  |
| *No* | 124 (33) | 294 (33) | 208 (34) | χ^2^ = 0.10 | 0.9496 | 32 (38) | 192 (34) | 402 (33) | χ^2^ = 1.05 | 0.5917 |
| Nausea (%) |  |  |  |  |  |  |  |  |  |  |
| *Yes* | 289 (77) | 732 （82） | 507 （82） |  |  | 67 (79) | 460 (81) | 1,001 (81) |  |  |
| *No* | 85 (23) | 161 （18） | 110 （18） | χ^2^ = 4.48 | 0.1065 | 18 (21) | 107 (19) | 231 (19) | χ^2^ = 0.31 | 0.8582 |
| Vomiting (%) |  |  |  |  |  |  |  |  |  |  |
| *Yes* | 254 （68） | 658 （74） | 457 （74） |  |  | 60 (71) | 412 (73) | 897 (73) |  |  |
| *No* | 120 （32） | 235 （26） | 160 （26） | χ^2^ = 5.33 | 0.0697 | 25 (29) | 155 (27) | 335 (27) | χ^2^ = 0.20 | 0.9060 |
| Photophobia (%) |  |  |  |  |  |  |  |  |  |  |
| *Yes* | 302 （81） | 700 （78） | 489 （79） |  |  | 69 (81) | 466 (82) | 956 (78) |  |  |
| *No* | 72 （19） | 193 （22） | 128 （21） | χ^2^ = 0.90 | 0.6384 | 16 (19) | 101 (18) | 276 (22) | χ^2^ = 5.18 | 0.0751 |
| Phonophobia (%) |  |  |  |  |  |  |  |  |  |  |
| *Yes* | 317 (85) | 759 (85) | 519 (84) |  |  | 73 (86) | 481 (85) | 1,041 (85) |  |  |
| *No* | 57 (15) | 134 (15) | 98 (16) | χ^2^ = 0.22 | 0.8958 | 12 (14) | 86 (15) | 191 (15) | χ^2^ = 0.14 | 0.9342 |
| Aggrevation by Physical Activity (%) |  |  |  |  |  |  |  |  |  |  |
| *Yes* | 343 (92) | 818 (92) | 566 (92) |  |  | 79 (93) | 526 (93) | 1,122 (92) |  |  |
| *No* | 31 (8) | 75 (8) | 51 (8) | χ^2^ = 0.01 | 0.9952 | 6 (7) | 41 (7) | 110 (8) | χ^2^ = 1.65 | 0.4373 |

Supplemental Table S6. Full lists of eQTL signals identified for SNP rs10456100 on *KCNK5*.

| Gene Symbol | SNP | *P*-Value | NES | T-statistic | Tissue |
| --- | --- | --- | --- | --- | --- |
| *KCNK5* | rs10456100 | 2.70×10^-10^ | -0.350 | -6.40 | Adipose - Subcutaneous |
| *KCNK5* | rs10456100 | 0.002 | -0.140 | -3.20 | Artery - Tibial |
| *KCNK5* | rs10456100 | 0.002 | -0.130 | -3.10 | Thyroid |
| *KCNK5* | rs10456100 | 0.003 | -0.140 | -3.00 | Stomach |
| *KCNK5* | rs10456100 | 0.005 | -0.120 | -2.80 | Adipose - Visceral (Omentum) |
| *KCNK5* | rs10456100 | 0.010 | -0.210 | -2.60 | Prostate |
| *KCNK5* | rs10456100 | 0.010 | -0.160 | -2.60 | Breast - Mammary Tissue |
| *KCNK5* | rs10456100 | 0.033 | -0.250 | -2.20 | Brain - Putamen (basal ganglia) |
| *KCNK5* | rs10456100 | 0.057 | -0.120 | -1.90 | Pancreas |
| *KCNK5* | rs10456100 | 0.092 | 0.088 | 1.70 | Esophagus - Mucosa |
| *KCNK5* | rs10456100 | 0.097 | 0.057 | 1.70 | Small Intestine - Terminal Ileum |
| *KCNK5* | rs10456100 | 0.11 | -0.064 | -1.60 | Lung |
| *KCNK5* | rs10456100 | 0.13 | 0.050 | 1.50 | Nerve - Tibial |
| *KCNK5* | rs10456100 | 0.14 | 0.110 | 1.50 | Liver |
| *KCNK5* | rs10456100 | 0.14 | -0.038 | -1.50 | Skin - Not Sun Exposed (Suprapubic) |
| *KCNK5* | rs10456100 | 0.16 | 0.150 | 1.40 | Brain - Cortex |
| *KCNK5* | rs10456100 | 0.22 | -0.046 | -1.20 | Esophagus - Muscularis |
| *KCNK5* | rs10456100 | 0.31 | -0.150 | -1.00 | Uterus |
| *KCNK5* | rs10456100 | 0.32 | -0.074 | -0.99 | Adrenal Gland |
| *KCNK5* | rs10456100 | 0.32 | 0.060 | 1.00 | Artery - Coronary |
| *KCNK5* | rs10456100 | 0.33 | -0.049 | -0.97 | Colon - Sigmoid |
| *KCNK5* | rs10456100 | 0.34 | -0.055 | -0.95 | Artery - Aorta |
| *KCNK5* | rs10456100 | 0.34 | -0.110 | -0.95 | Brain - Amygdala |
| *KCNK5* | rs10456100 | 0.37 | 0.120 | 0.90 | Cells - EBV-transformed lymphocytes |
| *KCNK5* | rs10456100 | 0.38 | 0.092 | 0.89 | Brain - Frontal Cortex (BA9) |
| *KCNK5* | rs10456100 | 0.39 | -0.110 | -0.87 | Brain - Spinal cord (cervical c-1) |
| *KCNK5* | rs10456100 | 0.42 | 0.087 | 0.80 | Brain - Caudate (basal ganglia) |
| *KCNK5* | rs10456100 | 0.43 | 0.052 | 0.80 | Heart - Left Ventricle |
| *KCNK5* | rs10456100 | 0.5 | 0.043 | 0.67 | Cells - Cultured fibroblasts |
| *KCNK5* | rs10456100 | 0.52 | -0.033 | -0.64 | Muscle - Skeletal |
| *KCNK5* | rs10456100 | 0.58 | 0.068 | 0.56 | Brain - Substantia nigra |
| *KCNK5* | rs10456100 | 0.59 | 0.031 | 0.54 | Testis |
| *KCNK5* | rs10456100 | 0.64 | -0.011 | -0.47 | Skin - Sun Exposed (Lower leg) |
| *KCNK5* | rs10456100 | 0.66 | -0.048 | -0.45 | Brain - Cerebellum |
| *KCNK5* | rs10456100 | 0.66 | -0.048 | -0.45 | Brain - Hippocampus |
| *KCNK5* | rs10456100 | 0.67 | 0.027 | 0.43 | Heart - Atrial Appendage |
| *KCNK5* | rs10456100 | 0.7 | -0.011 | -0.38 | Colon - Transverse |
| *KCNK5* | rs10456100 | 0.72 | 0.045 | 0.36 | Vagina |
| *KCNK5* | rs10456100 | 0.76 | -0.017 | -0.30 | Pituitary |
| *KCNK5* | rs10456100 | 0.77 | 0.024 | 0.29 | Minor Salivary Gland |
| *KCNK5* | rs10456100 | 0.84 | -0.019 | -0.21 | Ovary |
| *KCNK5* | rs10456100 | 0.93 | 0.003 | 0.08 | Whole Blood |
| *KCNK5* | rs10456100 | 0.96 | 0.006 | 0.05 | Brain - Hypothalamus |
| *KCNK5* | rs10456100 | 0.97 | 0.004 | 0.04 | Brain - Anterior cingulate cortex (BA24) |
| *KCNK5* | rs10456100 | 0.98 | -0.002 | -0.02 | Spleen |

NES: normalized effect size.

Supplemental Table S7. Full lists of eQTL signals identified for SNP rs7775721 on *FHL5*.

| Gene Symbol | SNP | *P*-Value | NES | T-statistic | Tissue |
| --- | --- | --- | --- | --- | --- |
| *FHL5* | rs7775721 | 0.0008 | -0.24 | -3.40 | Brain - Putamen (basal ganglia) |
| *FHL5* | rs7775721 | 0.0050 | -0.13 | -2.80 | Artery - Aorta |
| *FHL5* | rs7775721 | 0.0059 | -0.21 | -2.80 | Brain - Frontal Cortex (BA9) |
| *FHL5* | rs7775721 | 0.0100 | 0.18 | 2.60 | Brain - Cerebellum |
| *FHL5* | rs7775721 | 0.0120 | 0.16 | 2.50 | Vagina |
| *FHL5* | rs7775721 | 0.0210 | 0.14 | 2.30 | Prostate |
| *FHL5* | rs7775721 | 0.0220 | -0.08 | -2.30 | Lung |
| *FHL5* | rs7775721 | 0.0390 | -0.09 | -2.10 | Colon - Sigmoid |
| *FHL5* | rs7775721 | 0.0670 | -0.17 | -1.90 | Brain - Substantia nigra |
| *FHL5* | rs7775721 | 0.0830 | -0.05 | -1.70 | Adipose - Visceral (Omentum) |
| *FHL5* | rs7775721 | 0.0890 | 0.09 | 1.70 | Pancreas |
| *FHL5* | rs7775721 | 0.0890 | 0.11 | 1.70 | Uterus |
| *FHL5* | rs7775721 | 0.12 | -0.03 | -1.60 | Artery - Tibial |
| *FHL5* | rs7775721 | 0.12 | -0.10 | -1.60 | Brain - Hypothalamus |
| *FHL5* | rs7775721 | 0.13 | -0.03 | -1.50 | Testis |
| *FHL5* | rs7775721 | 0.19 | -0.12 | -1.30 | Brain - Anterior cingulate cortex (BA24) |
| *FHL5* | rs7775721 | 0.23 | -0.03 | -1.20 | Nerve - Tibial |
| *FHL5* | rs7775721 | 0.24 | -0.07 | -1.20 | Brain - Nucleus accumbens (basal ganglia) |
| *FHL5* | rs7775721 | 0.24 | 0.06 | 1.20 | Heart - Left Ventricle |
| *FHL5* | rs7775721 | 0.25 | 0.09 | 1.10 | Brain - Cerebellar Hemisphere |
| *FHL5* | rs7775721 | 0.26 | -0.05 | -1.10 | Artery - Coronary |
| *FHL5* | rs7775721 | 0.28 | -0.07 | -1.10 | Brain - Caudate (basal ganglia) |
| *FHL5* | rs7775721 | 0.32 | -0.03 | -0.99 | Breast - Mammary Tissue |
| *FHL5* | rs7775721 | 0.34 | 0.12 | 0.96 | Cells - EBV-transformed lymphocytes |
| *FHL5* | rs7775721 | 0.36 | -0.06 | -0.92 | Pituitary |
| *FHL5* | rs7775721 | 0.36 | 0.06 | 0.91 | Small Intestine - Terminal Ileum |
| *FHL5* | rs7775721 | 0.4 | 0.04 | 0.84 | Whole Blood |
| *FHL5* | rs7775721 | 0.46 | 0.03 | 0.75 | Esophagus - Muscularis |
| *FHL5* | rs7775721 | 0.46 | 0.04 | 0.75 | Heart - Atrial Appendage |
| *FHL5* | rs7775721 | 0.47 | 0.02 | 0.73 | Muscle - Skeletal |
| *FHL5* | rs7775721 | 0.48 | -0.02 | -0.71 | Adipose - Subcutaneous |
| *FHL5* | rs7775721 | 0.49 | -0.05 | -0.70 | Brain - Hippocampus |
| *FHL5* | rs7775721 | 0.64 | 0.03 | 0.47 | Adrenal Gland |
| *FHL5* | rs7775721 | 0.67 | -0.01 | -0.43 | Skin - Sun Exposed (Lower leg) |
| *FHL5* | rs7775721 | 0.71 | -0.01 | -0.37 | Colon - Transverse |
| *FHL5* | rs7775721 | 0.75 | -0.03 | -0.32 | Liver |
| *FHL5* | rs7775721 | 0.77 | 0.03 | 0.30 | Minor Salivary Gland |
| *FHL5* | rs7775721 | 0.79 | -0.01 | -0.27 | Thyroid |
| *FHL5* | rs7775721 | 0.84 | 0.02 | 0.20 | Spleen |
| *FHL5* | rs7775721 | 0.86 | 0.02 | 0.18 | Brain - Amygdala |
| *FHL5* | rs7775721 | 0.87 | -0.02 | -0.16 | Brain - Spinal cord (cervical c-1) |
| *FHL5* | rs7775721 | 0.91 | -0.008 | -0.12 | Cells - Cultured fibroblasts |
| *FHL5* | rs7775721 | 0.91 | -0.004 | -0.12 | Skin - Not Sun Exposed (Suprapubic) |
| *FHL5* | rs7775721 | 0.92 | -0.007 | -0.10 | Brain - Cortex |
| *FHL5* | rs7775721 | 0.94 | -0.003 | -0.07 | Esophagus - Mucosa |
| *FHL5* | rs7775721 | 0.94 | -0.004 | -0.08 | Ovary |
| *FHL5* | rs7775721 | 0.96 | 0.002 | 0.05 | Stomach |

NES: normalized effect size.


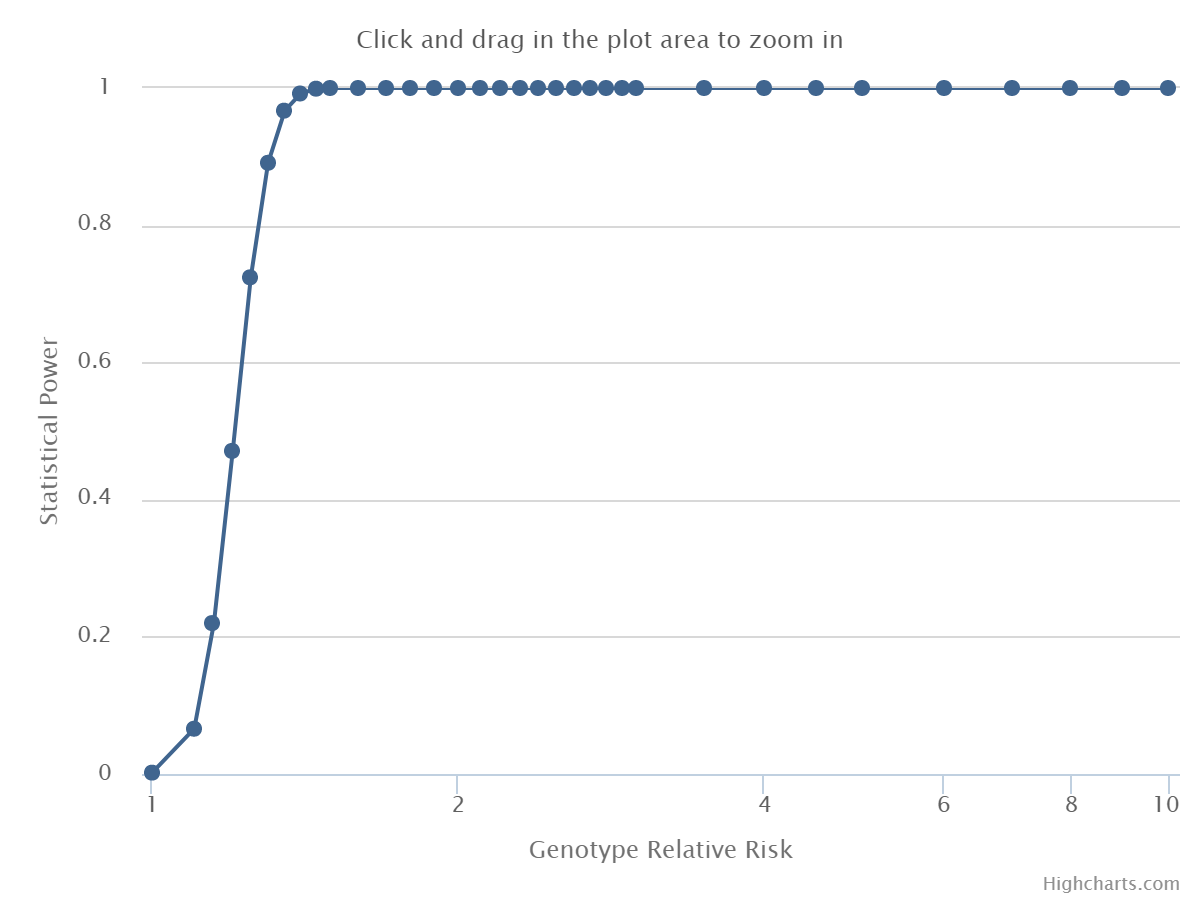


82.8%, RR=1.2

Supplemental Figure S1. Statistical power achieved by the study setting versus genotypic relative risk. The figure was generated by GAS power calculator (version 4.2.5, http://csg.sph.umich.edu/abecasis/gas_power_calculator/)


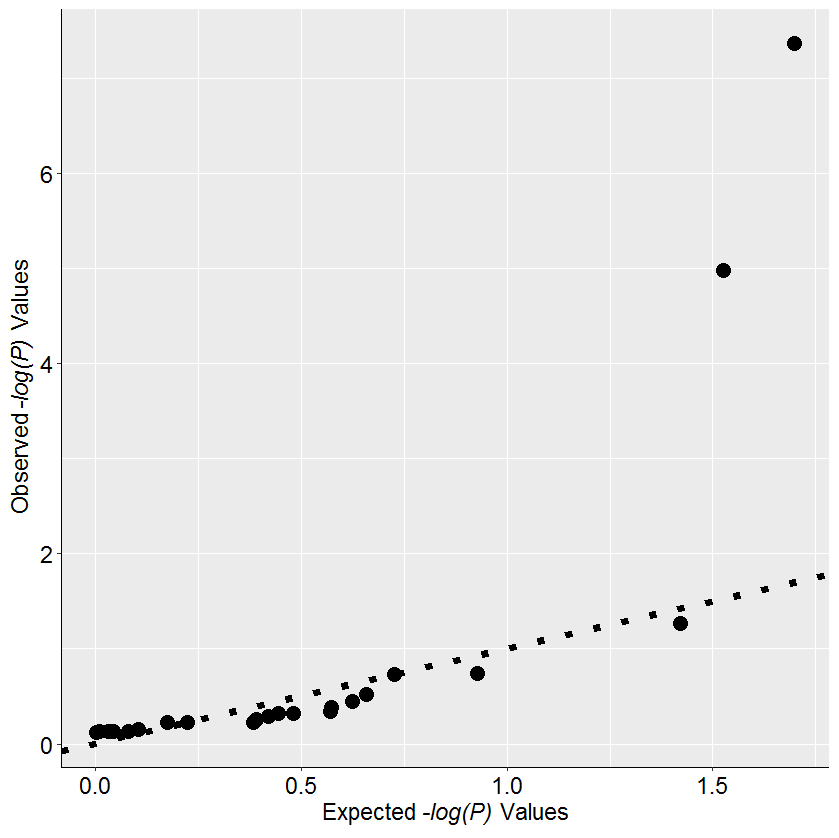


Supplemental Figure S2. Q-Q plot for the allelic association signals for each SNP.
